# Spatial analysis of genetic clusters and epidemiologic factors related to wild poliovirus type 1 persistence in Afghanistan and Pakistan

**DOI:** 10.1371/journal.pgph.0000251

**Published:** 2022-06-13

**Authors:** Amalia Mendes, Ari Whiteman, Kelley Bullard, Salmaan Sharif, Adnan Khurshid, Muhammad Masroor Alam, Muhammad Salman, Vanessa Ford, Taisha Blair, Cara C. Burns, Derek Ehrhardt, Jaume Jorba, Christopher H. Hsu

**Affiliations:** 1 Centers for Disease Control and Prevention, Atlanta, Georgia, United States of America; 2 DRT Strategies Inc., Arlington, Virginia, United States of America; 3 Peraton, Atlanta, Georgia, United States of America; 4 IHRC Inc., Atlanta, Georgia, United States of America; 5 Department of Virology, National Institute of Health, Islamabad, Pakistan; 6 Department of Pediatrics, Emory University, Atlanta, Georgia, United States of America; Swiss Federal Institute of Technology Zurich: Eidgenossische Technische Hochschule Zurich, SWITZERLAND

## Abstract

Following the certification of the World Health Organization Region of Africa as free of serotype 1 wild poliovirus (WPV1) in 2020, Afghanistan and Pakistan represent the last remaining WPV1 reservoirs. As efforts continue in these countries to progress to eradication, there is an opportunity for a deeper understanding of the spatiotemporal characteristics and epidemiological risk factors associated with continual WPV1 circulation in the region. Using poliovirus surveillance data from 2017–2019, we used pairwise comparisons of VP1 nucleotide sequences to illustrate the spatiotemporal WPV1 dispersal to identify key sources and destinations of potentially infected, highly mobile populations. We then predicted the odds of WPV1 detection at the district level using a generalized linear model with structural indicators of health, security, environment, and population demographics. We identified evidence of widespread population mobility based on WPV1 dispersal within and between the countries, and evidence indicating five districts in Afghanistan (Arghandab, Batikot, Bermel, Muhamandara and Nawzad) and four districts in Pakistan (Charsada, Dera Ismail Khan, Killa Abdullah and Khyber) act as cross-border WPV1 circulation reservoirs. We found that the probability of detecting WPV1 in a district increases with each armed conflict event (OR = 1·024, +- 0·008), level of food insecurity (OR = 1·531, +-0·179), and mean degrees Celsius during the months of greatest precipitation (OR = 1·079, +- 0·019). Our results highlight the multidisciplinary complexities contributing to the continued transmission of WPV1 in Afghanistan and Pakistan. We discuss the implications of our results, stressing the value of coordination during this final chapter of the wild polio virus eradication initiative.

## Introduction

Wild poliovirus types 1, 2, and 3 (WPV1, WPV2, WPV3) cause poliomyelitis (polio), a paralytic disease that can lead to lifelong disability and death [1]. WPV1 remains endemic in only Afghanistan and Pakistan, where cross-border transmission has been well documented [2]. The Global Polio Eradication Initiative (GPEI) established a target of >90% population immunity in Pakistan to interrupt WPV transmission [3, 4]. As of 2021, the only two endemic countries remaining, reported the lowest number of WPV1 cases (one in each country) in a single year. Prior to that, the two countries reported huge resurgences in the number of cases, particularly post 2017 [5]. In 2019, 147 WPV1 cases were reported in Pakistan, a nearly 12-fold increase over 2018 [6]. Similarly, twenty-nine cases were reported in Afghanistan in 2019, compared to 21 in 2018 [2]. Numerous factors have been documented or hypothesized as reasons for continued WPV1 persistence in the region, including difficulty in accessing remote populations, insecurity due to insurgency at or near shared borders affecting supply chains, and increased violence targeting healthcare workers and their protective security personnel [4].

**Fig 1:**
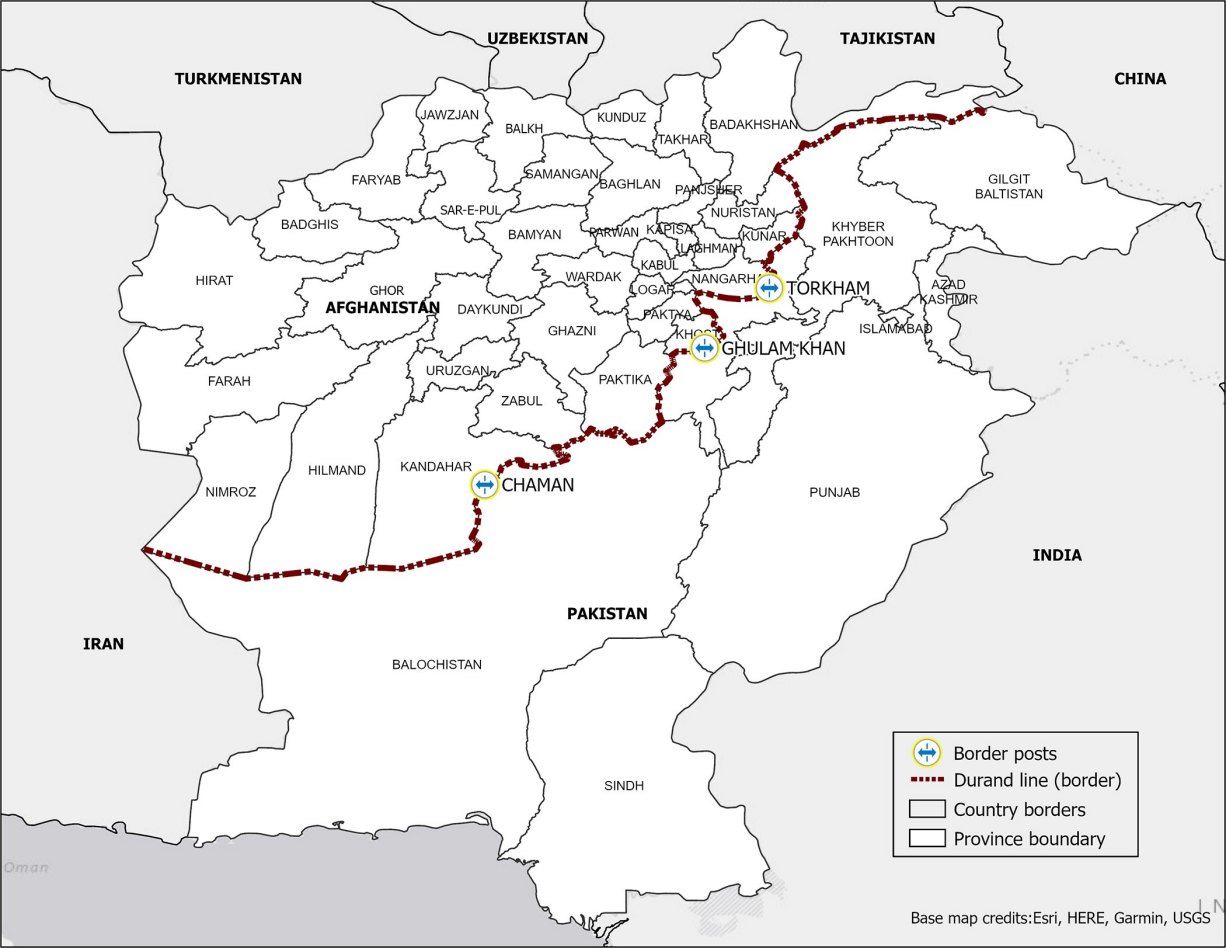
Official border crossings (border posts) between Afghanistan and Pakistan. Base map service credits- Esri, HERE, Garmin, (c) OpenStreetMap contributors, and the GIS user community [8].

High population mobility is another major cause of concern of regional viral spread. The Durand line, the demarcation between Afghanistan and Pakistan, is a highly porous border. There are three formal crossings (Fig 1), which include Torkham and Chaman, both accessed by mobile ethnic groups, the Pashtuns and Balochis [7], plus eight informal crossings available by vehicle, and an additional 235 crossings that are navigable only by foot or animal.

Prior to the tightening of border control measures in late 2014, it is estimated that approximately 20,000 persons were crossing the Durand line daily at Torkham [7]. Chaman remains unrestricted with estimates up to 30,000 crossings daily. Furthermore, both border crossings have erratic border management policies. Despite administrative differences, crossings are widespread, causing the two countries to function as a single epidemiological block [9].

As efforts continue to manage and implement activities needed to interrupt WPV1 transmission in both countries, high quality poliovirus surveillance is critical to understand where transmission occurs and what factors contribute to viral circulation. Surveillance efforts provide the time, space and phylogenetic (i.e., gene analysis of sewage specimens) data necessary to describe a site as a source or sink. Specifically, sources tend to exhibit more localized and short-term case detection, in contrast to sinks which are more often the location of repeated case detection over time and may be larger in area as well. Phylogenetics can further support source or sink designation by illustrating spatiotemporal mismatch between two samples, assuming a constant rate of nucleotide substitution. For example, two samples from distant districts with few relative nucleotide changes may indicate more recent or direct migration from one district to the other, whereas two samples from adjacent districts with high relative nucleotide changes may indicate local transmission that is going undetected by surveillance efforts. Thus, surveillance quality is integral to determining the spatiotemporal migration of infected persons.

Identifying common sources and sinks of mobile populations can provide health authorities with the information to potentially focus limited resources on areas with particularly high numbers of at-risk individuals. Thus, our study had two objectives. Firstly, we conducted a phylogenetic analysis of WPV1 isolated exclusively from fecal specimens collected in case investigations of acute flaccid paralysis (AFP) during 2017–2019. By linking affected districts with WPV1 lineages, we sought to map WPV1 circulation within and between the two countries over time to visualize and describe transborder movement. Prior studies [10] employing similar methodologies have been conducted, however our study infers transmission patterns from genetic relationships among isolates in the context of population movement across the Afghanistan-Pakistan border. Secondly, we sought to examine the epidemiological characteristics that could drive some districts to exhibit higher risk of WPV1 transmission than others based on several structural estimators of potential health importance.

## Methods

### Data synthesis and variable justification

Laboratory testing of specimens collected during poliovirus surveillance in Pakistan and Afghanistan is conducted by the Department of Virology of the National Institute of Health in Islamabad (Pakistan), a WHO Regional Reference Laboratory (RRL). Virus isolation and nucleotide sequencing of the complete viral protein 1 (VP1) capsid coding region (906 nucleotides) were performed following the methods of the Global Poliovirus Laboratory Network [11–13]; sequence data are routinely analyzed [14]. We used reported AFP surveillance WPV1 data from RRL-Pakistan for Pakistan and Afghanistan with paralysis specimen dated during 2017–2019. AFP, a clinical syndrome, is a collection of symptoms defined as the sudden onset of weakness/floppiness in any part of the body in a child under the age of 15 years. AFP can have many infectious and non-infectious causes with polio being one of them [15]. Sequence data from environmental surveillance (ES) were excluded from this study, as ES sequences have high probability of being from multiple persons.

For the predictive model of WPV1 detection, we used sub-national data from a range of sources (Table 1). We focused our selection of independent variables on topics with either previously confirmed or hypothesized associations to vaccine-preventable disease. The number of health facilities and food insecurity may be representative of general health infrastructure in the district and have previously been linked to the presence of various preventable diseases [16]. Mean temperature of the wettest quarter, mean temperature of the driest quarter, precipitation seasonality, and precipitation of the wettest quarter are four climatic variables that have previously associated with the presence of water and sewage-borne pathogens in regions with unsealed or open sanitation systems [17]. Number of conflicts was a proxy of security in a district, which can be a key barrier to the vaccination campaigns and the maintenance of other key services [18]. We used disaggregated data available through the armed conflict location and event data (ACLED) project for Pakistan and an openly available source developed by the National Police Command Center (NPCC) for Afghanistan. Lastly, status as a border or non-border located district, presence of border-crossing roads, and median slope of the terrain are each focused on conditions along the Durand Line, where we hypothesize that sustained transmission may be associated with the mobility of border-located populations and convenience of traversing a mountainous region.

**Table 1:** Data sources.

Model Input	Pakistan		Afghanistan		Unit of Analysis	Limitations
	Source	Year	Source	Year		
Genetic Clusters	NIH-Pakistan/CDC	2017–2019	NIH-Pakistan/CDC	2017–2019	Groups of WPV1 isolates sharing ≥95% sequence identity	Surveillance sensitivity
Detection of WPV1	POLIS	2019	POLIS	2019	Number of reported WPV 1 cases	Possible missing cases due to poor localized surveillance quality
Health Facilities ^a^	WHO, Health Cluster Partners, OSM	2018, 2020	OSM	2020	Number of health facilities recorded during health resource mapping assessments and volunteered collection of geographic information (Min: 0 | Max: 247)	Limited coverage of health facilities per district during assessment with potential inaccuracies within crowdsourced data collected
Food Insecurity ^b^	WFP	2020	WFP	2007–2018	Food insecurity level (1 = low, 2 = medium, 3 = high, 4 = very high)	Discrepancy in years between Afghanistan and Pakistan
Mean temperature of the wettest quarter	Worldclim	Aggregate 1970–2000	Worldclim	Aggregate 1970–2000	Degrees Celsius (Min: -10 | Max: 34)	Temporal mismatch
Mean temperature of Driest Quarter	Worldclim	Aggregate 1970–2000	Worldclim	Aggregate 1970–2000	Degrees Celsius (Min: -15 | Max: 33)	Temporal mismatch
Precipitation Seasonality (Coefficient of Variation)	Worldclim	Aggregate 1970–2000	Worldclim	Aggregate 1970–2000	Coefficient of variation (Min: 41 | Max: 163)	Temporal mismatch
Precipitation of Wettest Quarter	Worldclim	Aggregate 1970–2000	Worldclim	Aggregate 1970–2000	Millimeters (Min: 35 | Max: 679)	Temporal mismatch
Population Density ^c^	Worldpop	2019	Geopode, Novel-t	2017	10^th^ percentile groups: persons per square-kilometer (Min: 0 | Max: 59,369)	Discrepancy in years and assessment types for Afghanistan and Pakistan
Conflicts ^d^	Armed Conflict Location & Event Data Project (ACLED)	2019^d^	National Police Command Center, open sources	2019^e^	Number of conflict events (Min: 0 | Max: 88)	Varying categories for each dataset used. Reporting mechanisms differ for each source and either source could potentially underreport
Border Located	Calculated	-	Calculated	-	Binary (1 = border located district, 0 = non-border located district)	-
Median slope of terrain	DivaGIS		DivaGIS		Angular degrees (Min: 0 | Max: 20)	-
Cross border Road Access	OSM	2020	OSM	2020	Binary (1 = districts with roads crossing the border, 0 = districts without roads crossing the border)	Possibility of overlooked roads due to hilly terrain

^a^ This dataset uses a combination of two sources: 1. Health Resources and Services Availability Monitoring System (HeRAM) assessments during different emergencies in Pakistan, 2. Open Street Mapping (OSM). Included type of health facilities include Basic Health Units (BHUs), Rural Health Clinic (RHC), Family Assistance Program (FAP), clinics, Doctors, hospitals.

^b^ This dataset contains information on risk of food insecurity according to the Integrated Context Analysis (ICA). Poverty data was used as a proxy for vulnerability to food insecurity due to incomplete datasets available at national level. The Multi-Dimensional Poverty Index (MPI), was the main indicator used for the analysis, measuring persons experiencing simultaneous acute deprivations in health, education, and living standards. To account for missing districts in part of FATA and Gilgit Baltistan provinces, we used the WFP In-depth Food Security and Livelihood Assessments from 2014 & 2017, a cross-sectional household survey. The assessment included household, market and focus group discussions covering consumption based coping strategies, household expenditure, market capacity and supply, community profile, population movement, etc.

^c^ This dataset is a resultant of predictive modelling using spatial regression models that includes the latest data from the Socio-Demographic and Economic Surveys (SDES), additional small area surveys, UN population division estimates and ancillary geospatial covariate data associated with variation in population density.

^d^ Disaggregated data based on daily news articles. Categories strategic developments, battles, violence against civilians, riots, explosives/remote violence, protests

^e^ Aggregated data based on daily news articles. Categories include civil unrest, explosive hazard, hostile action, law enforcement, Military

### Data analysis

Sequence analysis, as reported by RRL-Pakistan, includes classification of WPV1 sequences in genetic clusters (clusters are defined as groups of isolates sharing ≥95% sequence identity in the VP1 coding region). Genetic clusters are used as a surveillance mechanism to track progress towards polio eradication by confirming detection, identifying origin and tracking the geographic spread at the regional and local level [19]. From 2017–2019 cluster data, two distinct genetic clusters (R4B5C5B2B- 81 isolates and R4B5C4C2- 36 isolates) were selected based on inferred evidence of cross-border transmission using comparative sequence analysis of the VP1 capsid region (906 nucleotides) of WPV isolates detected from AFP surveillance. The comparative analysis includes inference of the phylogenetic relationships among WPV isolates based on specimen dates and geographic detection of the isolate.

**Fig 2:**
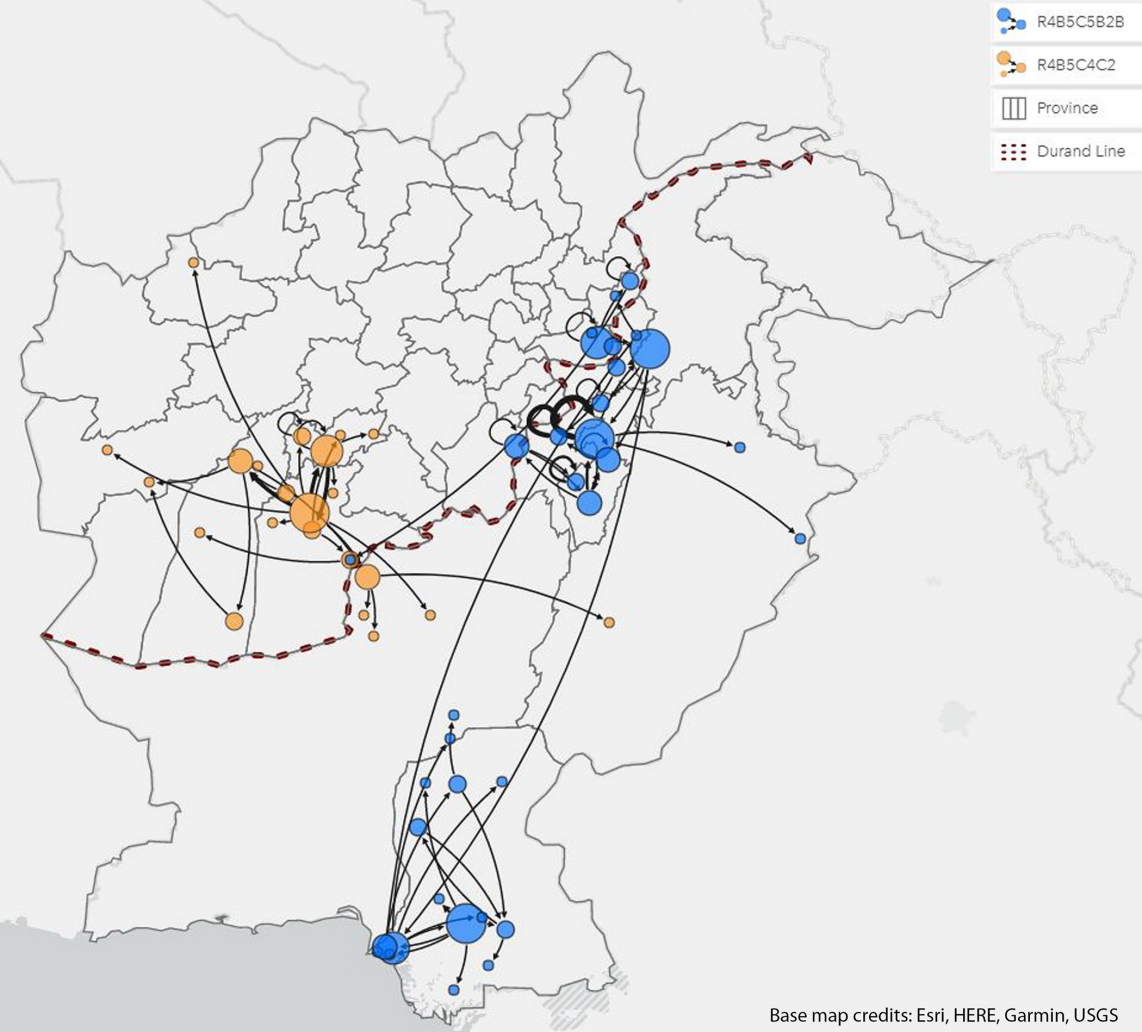
**Inferred pattern of transmission of WPV1 in genetic clusters R4B5C4C2 (yellow) and R4B5C5B2B (blue).** Base map service credits- Esri, HERE, Garmin, (c) OpenStreetMap contributors, and the GIS user community [8]. Note- A. Proportional symbols depicted as circles, indicate the number of closest identity matches an original Viral Protein 1 (VP1) sourced. B. The arrows are indicative of locations where the closest identity matches an original VP1 sourced. C. The arrows are indicative of locations where the closest identity match to a sequence was identified and does not represent directionality. D. The thickness of each arrow measures the influence of a node.

**Fig 3:**
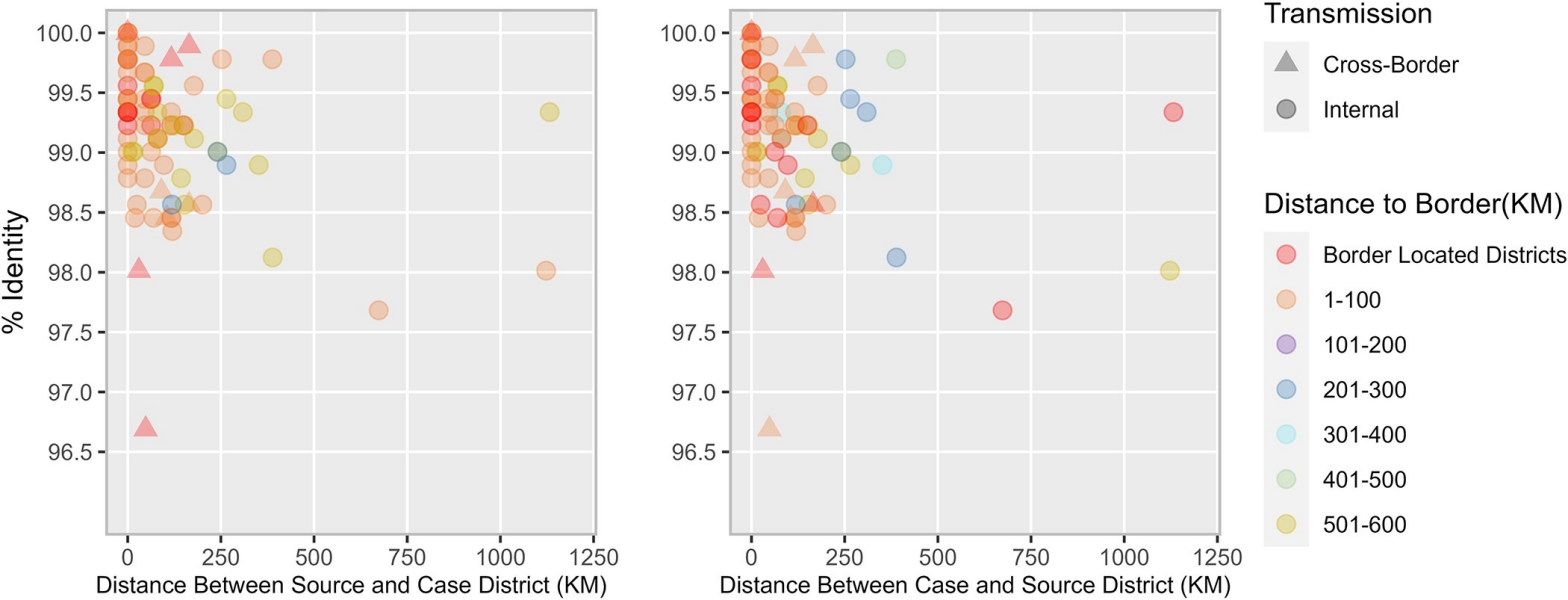
Scatterplot depicting A. nucleotide sequence identity (y-axis) for source districts in relation to the distance from its case district (x-axis) and distance from the border (color-coded) and B. nucleotide sequence identity (y-axis) for case districts in relation to the distance from its source district (x-axis) and distance from the border (color-coded) categorized under cluster R4B5C5B2B.

**Fig 4:**
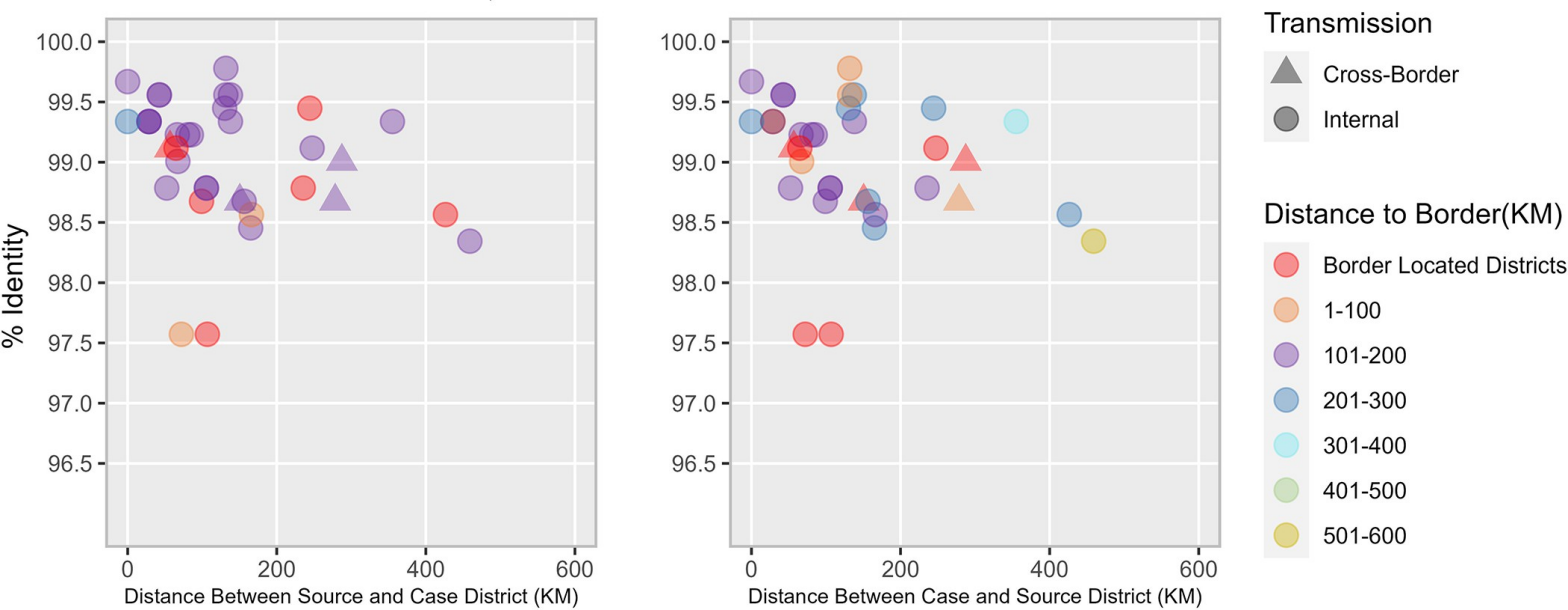
Scatterplot depicting A. nucleotide sequence identity (y-axis) for source districts in relation to the distance from its case district (x-axis) and distance from the border (color-coded) and B. nucleotide sequence identity (y-axis) for case districts in relation to the distance from its source district (x-axis) and distance from the border (color-coded) categorized under cluster R4B5C4C2.

Nucleotide alignments for each genetic cluster were obtained using the bioinformatics software Geneious^®^ Prime (Biomatters Limited, Auckland, New Zealand) version 2020·2·4 and subsequently pairwise comparisons of sequence identity (number of identical VP1 sites between two sequences over the total length of VP1 [906 nucleotides]) were obtained using functions within Geneious. The sequence identity matrix was ordered according to specimen date of the corresponding AFP case and the closest match preceding the specimen date for each case was recorded and geographically linked as shown in Figs 2–4. The map shown in Fig 2 was built in ArcGIS Insights, version 2020·3·0·3150 (ESRI, Redlands, California, United States of America). The nodes presented in Fig 2, representing isolates identified within districts, are sized by graduated symbols using a method called centrality [20]. The centrality type chosen for this map employs an outdegree centrality which refers to the number of outgoing links to which a district contributes. Thus, the larger the proportional symbol, the more identity matches an original VP1 sourced. Additionally, the thickness of each arrow measures the influence of a node. To understand the relationship between the nucleotide sequence percent identity for source districts in relation to the distance from its case district, an ordinary least squares regression was conducted for both clusters. This was intended as a simple measure of surveillance quality, where a weak association would imply considerable surveillance gaps (i.e., high proximity cases with limited percent identity, meaning persistent localized transmission without being detected by surveillance systems).

For the predictive model, we employed a generalized linear model fitted with a binomial distribution, with our outcome, presence of WPV1 in 2019, being dichotomous. We transformed the outcome variable into binary format in order to reduce the effect of inconsistent surveillance and monitoring among districts. One district, in Afghanistan, had over 100 conflict events and therefore produced an unreliable value with the potential for skewing estimates so was excluded from the analysis. We present the model estimates as odds where a one unit increase in an independent variable, accounts for a subsequently increased or decreased odds of the detection of WPV1, controlling for the other confounders. We conducted the models in R version 1·2·5033 (R Core Team, Vienna, Austria).

## Results

### Genetics

The mean nucleotide sequence pairwise identities for the R4B5C5B2B and R4B5C4C2 sub-genotypes were 99·15% (range: 96·68% - 100%) and 99·01% (range: 97·57% - 99·77), respectively. Both genetic clusters exhibit a weak negative association between identity match percentages and distance between case and source districts indicating sub-optimal surveillance (Fig 3, Cluster R4B5C5B2B: R2 = 0.072, P = 0·11, SE = 0·0007; Fig 4, Cluster R4B5C4C2: R2 = 0·083, P = 0·008, SE = 0·0003).

For cluster R4B5C5B2B, 62% (51) of all 81 cluster R4B5C5B2B isolates shared the closest match (according to specimen date of the corresponding AFP case and the closest match preceding the specimen date) to Pakistan’s (P) Khyber Pakhtunkhwa (KP) province, with Bannu (P) having the highest number of closest identity matches (outdegree centrality 0.21) with 99%-100% nucleotide sequence identity followed by Charsada (P) and Batikot in Afghanistan (A), (outdegree centrality 0·18 and 0·15 respectively). The remaining isolates shared the closest match to Sindh (P) (25%, 20), Nangahar (A) (7%, 6), Paktika (A) (4%, 3) and Kunar (A) (2%, 2). Six cross-border transmissions were linked to Nangahar (A) (2) and Paktika (A) (2) and Khyber Pakhtukhwa (P) (2) province and were observed to be concentrated in the northern epidemiologic block for both countries (Batikot, Muhmandara and Bermel in Afghanistan and Charsada, and Dera Ismail Khan in Pakistan). The two cross-border transmissions linked to Charsada (P) and Dera Ismail Khan (P) shared the closest match to Watapur (A) and Bermel (A) district.

The sequence data indicated cluster R4B5C5B2B to be more widely distributed in the bordering areas and southern areas of Pakistan than cluster R4B5C4C2. The six cross-border WPV1 strains in cluster R4B5C5B2B appeared to share the closest matches to districts in the northern reservoir and were observed to be 0–58 km away from the border with a nucleotide sequence identity ranging from 96·68% - 100%.

Cluster R4B5C4C2 contributed to four cross-border transmissions (Fig 2), three of which shared closest matches to isolates sourced to Afghanistan and later identified in Pakistan. Two were sourced from Arghandab (A) district in Kandahar province and one was sourced from Nawzad (A) district in Hilmand province. Among the 36 R4B5C4C2 WPV1 cluster isolates, 53% (19) shared closest matches to the province of Kandahar (A), 22% to Uruzgan (A) (8), 14% to Hilmand (A) (5) and 11% to Balochistan (P) (4). Upon further examination, 39% (14) of all 36 isolates in cluster R4B5C4C2 group shared closest matches to Arghandab (A) district (outdegree centrality of 0·47) located in Kandahar province, 100 km from the Durand line, with a nucleotide sequence identity ranging from 98·33%– 99·55%. This was followed by Tirinkot (A) (7 cases, outdegree centrality of 0·23), Nawzad (A)and Killa Abdullah (P) (4 cases each, outdegree centrality of 0.19), Kandahar(A) and Spinboldak (A) (2 cases each, outdegree centrality of 0·09) and Garmser (A), Ghorak (A) and Shahid-e-Hassas (A) (1 case each, outdegree centrality of 0·047).

The scatterplot for cluster R4B5C4C2 (Fig 4) demonstrated 78% (7) source districts and 65% (15) case districts to be more than 100 km away from the Durand line as compared to cluster R4B5C5B2B which exhibited 72% (18) source and 91% (31) case districts located on or within 1–100 km of the border. Three of the four cross-border isolates for this cluster were located more than 100 km away from the Durand line, except for a single cross-border transmission originally identified in the district of Killa Abdullah (P), located at the border. The importation of four R4B5C4C2 WPV1 strains through cross-border transmissions all appeared to have been linked to districts in the southern reservoir with a nucleotide sequence identity ranging from 98·67% - 99·11%.

### Probability of WPV1 detection

**Fig 5:**
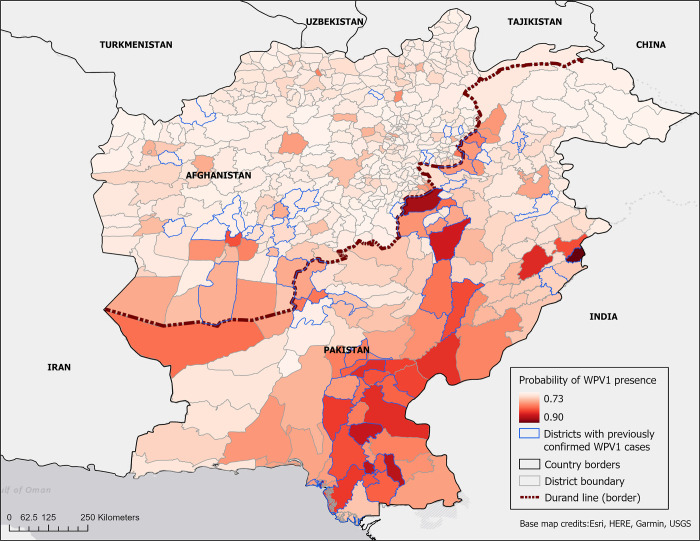
Probability of WPV1 presence in Afghanistan and Pakistan based on results of generalized linear model.

Districts with the highest odds of WPV1 detection were distributed throughout central and southern Pakistan and southern Afghanistan (Fig 5); however, of the 50 districts with the highest predicted probability of WPV1 detection, six were in Afghanistan. The three districts with the highest odds overall, all in Pakistan, include two highly urbanized areas, Lahore and Karachi, and one border district, Waziristan. The regions of southern Pakistan and the eastern portion of the Durand Line also included the most districts with high relative probabilities of WPV1 detection that have not actually reported WPV1 cases.

Controlling for other covariates, three independent variables were associated with significant (P < 0·05) increases in the odds of WPV1 detection in a district (Table 2), with no collinearity observed. First, each additional conflict event was associated with a 2·4% (OR = 1·024 ± 0·008) increase in the probability of WPV1 detection. Second, each increased level of food insecurity was associated with a 53·1% (OR = 1·531 ± 0·179) increase in the odds of WPV1 detection. Lastly, each increase in temperature during the wettest quarter of the year was associated with a 7·9% (OR = 1·079 ± 0·019) increase in the odds of WPV1. The remaining independent variables were not found to be associated with a significant change in the odds of WPV1 detection.

**Table 2:** Results of a generalized linear model predicting the odds of WPV1 detection in districts in Afghanistan and Pakistan.

	Odds Ratio	Std. Error	*P*
**Conflict events**	1·024	0·008	<0·01
**Food insecurity**	1·531	0·179	0·01
**Mean temperature of the wettest quarter**	1·079	0·019	<0·01
**Mean temperature of driest quarter**	1·055	0·040	0·17
**Precipitation seasonality (Coefficient of Variation)**	1·011	0·007	0·14
**Precipitation of wettest quarter**	0·998	0·002	0·18
**Median slope of terrain**	1·115	0·067	0·10
**Presence of cross-border roads**	1·484	0·890	0·65
**Border district**	3·959	0·848	0·10
**Number of health centers**	1·008	0·005	0·09
**Population density**	1·060	0·068	0·39

## Discussion

Our study illustrates a complex scenario of WPV1 transmission in Afghanistan and Pakistan. We found evidence of considerable human domestic and international migration in our analysis focused on two genetic clusters. The mobility of the populations residing in this region presents a key hurdle to reaching WPV1 eradication, as the dispersal of infected individuals has the potential to introduce the pathogen to many under-vaccinated communities. One additional complicating factor is the relatively low correlation between genetic identity and distance between case and source district, indicating that in many cases extended genetic divergence is found in high proximity cases. This extended divergence between geographically close cases may indicate limited or poor surveillance in the area, which failed to identify the cases closer to the time of their arrival and may be missing instances of local transmission. This underscores the need for high-quality surveillance systems in all districts that can detect possible cases in a timely manner. Additionally, because of the high porosity of the Durand Line and demonstrated examples of closely related samples found on both sides of the border, enhancing already existing data sharing agreements and surveillance cooperation between Afghanistan and Pakistan may substantially improve the efficacy of the currently fragmented system.

Understanding motives for migration is equally important. A survey conducted at the formal border-crossing at Torkham in June 2017, reported the most cited reasons for border-crossing included medical treatment (33%), lack of economic opportunity (22%), family (16%), and security (15%) [21]. Given the current security situation in the region, increased cross-border movement, internal displacement, and refugee crises may exacerbate already heightened concerns for viral circulation. As has been done in other regions of high mobility populations [21–24], administering both quantitative and qualitative surveys designed to understand the culture, ethnicity, demographics, spatiotemporal movement patterns, and vaccination status is a crucial first step to providing mobile populations with needed health services. Our results indicate that several districts, namely Argandhab, Tirinkot and Nawzad in Afghanistan and Killa Abdullah, Bermel, Dera Ismail Khan, Muhamandara and Charsada in Pakistan, may be common sources of transborder migration, and thus may serve as an effective hub for conducting population surveys in addition to focused vaccine campaigns as migration increases.

In light of the complicated consequences of human mobility on controlling WPV1 in the region, our model of WPV1 detection probability may provide clarity as to which districts may be at heightened risk. Armed conflict has been associated with a spectrum of poor health outcomes globally, including increases in childhood mortality [25, 26], reductions in vaccination coverage [27], increase in vaccine-preventable diseases, and even increases in cancer incidence [28]. Conflict events have inhibited the maintenance of health infrastructure often at the expense of healthcare and aid workers (including polio frontline workers) safety [29, 30]. In particular, reduced vaccination rates were observed up to 12 months after a security incident in Pakistan [4], while insurgent groups have banned house-to-house vaccination campaigns in areas of Afghanistan since April 2018 [31]. Effective vaccination administration may need to include specialized training approaches [32] and capacity building techniques that have been utilized successfully in conflict zones elsewhere.

Food insecurity is another concern globally and has been associated with infectious disease outbreaks through several different mechanisms [16]. Our results indicate that across both countries, food insecurity is associated with substantially increased probability of WPV1 detection, though the mechanism is unclear. Malnutrition has been found to reduce the immunogenicity of oral poliovirus vaccines in Pakistan [33] and elsewhere [34], while; chronically malnourished infants are at a heightened risk of WPV infection compared to healthy, even vaccinated, infants. Assessing these issues and expanding food access in Afghanistan and Pakistan is a difficult, key challenge to the eradication of WPV1 in the region.

Lastly, our study indicated the influence of warm, wet seasons in increased detection of poliovirus, an epidemiologic characteristic for general infectious disease transmission. The projected impacts of climate change in the Eastern Mediterranean and Middle East include considerable increases in median temperature and decreases in per capita water resources, which will burden already fragile environmental, agricultural, economic, and health systems [35]. While little is known about the effects of climate on poliovirus transmission, testable theories such as increased likelihood of contaminated water use during warm and wet periods and increases in diarrheal disease may be useful lines of future investigation.

Key limitations to our study may be addressed in future inquiries. First, most of our analysis and data collection was conducted right at the start of the COVID-19 pandemic and prior to the Taliban reestablishing their rule in Afghanistan following the withdrawal of U.S. troops and their NATO partners in mid-2021[36, 37]. Therefore, our study does need to be viewed within the context of these two significant political events. Second, this study focused on genetic clusters with identified cross-border case matches and did not include data from environmental samples. Other clusters that were contained domestically or occurred in different years may exhibit different spatiotemporal characteristics than what we observed. Third, though the surveillance systems in Afghanistan and Pakistan are very thorough and represent some of the most extensive polio surveillance systems globally, security and geographic challenges can cause surveillance quality to differ by province/districts within a country. This may incur sample bias in the predictive model due to the possible occurrence of undetected cases. Further, differences in the methods used to collect data on the same variables in different countries may have incurred geographic bias and temporal mismatch between the dates of collection and WPV1 detection. Fourth, the climate data we utilized is not current. Beyond basic temperature or precipitation averaged across the entire year, current data on sub-annual climate like temperature during the wettest quarter of the year, is not available. In a region with such significant seasonal weather swings, we chose not to use annual temperature or precipitation data, which despite being more current, may obscure the pathogenic qualities of certain areas during certain times of the year. Fifth, for the predictive model on WPV1 detection probability, there is likely heterogeneous error across the study area. While spatial models are traditionally used to account for this, the outcome was too rare for model convergence using several spatial analysis methods. We recognize that a non-spatial model may incur over or under-fit in some districts and advise that readers interpret the output of the predictive model with appropriate caution. While changes to these variables may have occurred since data was collected, these were unlikely to have been great enough to considerably alter the effects or lack of effect identified in the model. Lastly, when comparing district-level independent variables to individual-level disease outcomes, as we have done, the associations must always be viewed as correlational in nature. Finer scale data, both spatially and temporally, on possible estimators is needed to establish more causal linkages to WPV1 cases.

## Conclusion

While we look toward the total eradication of WPV1 from its last remaining zones of sustained transmission, our study highlights the multi-factorial complexities that underscore the importance of a multi-disciplinary approach to overcome the final challenges of WPV1 eradication. Human migration, armed conflict, malnutrition, and climate are interactive in cause and far-reaching in effect, though unlike WPV1 transmission, they are certainly not issue’s specific to Afghanistan and Pakistan. Thus, international and community collaborations will be key to overcoming these challenges, with evidence of successful programs employed elsewhere utilized as frameworks for interventions in the region. The historic efforts that led to the eradication of WPV2 and WPV3 showcase the impact of strong partnerships.

## Supporting information

S1 Data GLM variables for Afghanistan. (XLSX)

S2 Data GLM variables for Pakistan. (XLSX)

S3 Data Data dictionary describing GLM variables. (XLSX)

S4 Data Genetic sequence data for cluster R4B5C4C2_. (XLSX)

S5 Data Regression, source and case districts, cluster R4B5C4C2. (XLSX)

S6 Data Genetic sequence data for cluster R4B5C5B2B. (XLSX)

S7 Data Regression, source and case districts, cluster_R4B5C5B2B. (XLSX)

## Abbreviations

ACLED: Armed conflict location and event data
AFP: Acute flaccid paralysis
BHU: Basic Health Unit
CDC: Centers for Disease Control and Prevention
ES: Environmental surveillance
FAP: Family Assistance Programs
ICA: Integrated Context Analysis
MPI: Multi-Dimensional Poverty Index
NIH-Pakistan: National Institute of Health, Islamabad
NPCC: National Police Command Center
OR: Odd Ratio
OSM: Open Street Maps
RHC: Rural Health Centers
RRL: Regional Reference Laboratory
VP1: Viral protein 1
WPV1: Wild Polio Virus, type 1
WFP: World Food Programme
WHO: World Health Organization
